# *catena*-Poly[[tetra-μ_3_-isonicotinato-μ_3_-oxalato-μ_2_-oxalato-disamarium(III)disilver(I)] dihydrate]

**DOI:** 10.1107/S1600536809054208

**Published:** 2009-12-24

**Authors:** Zhao-yang Li, Shan-tang Yue

**Affiliations:** a School of Chemistry and Environment, South China Normal University, Guangzhou 510006, People’s Republic of China

## Abstract

In the title compound, {[AgSm(C_6_H_4_NO_2_)_2_(C_2_O_4_)]·H_2_O}_*n*_, the asymmetric unit contains one Sm^III^ ion, one Ag^I^ ion, two unique isonicotinate (ina) ligands, two half oxalate (ox) ligands (one on an inversion centre, the other on a twofold axis) and one uncoordinated water mol­ecule. The central Sm^III^ ion is nine-coordinated by four O-donor atoms from separate bidentate bridging ox ligands and five O-donor atoms from the two ina ligands (both bidentate) and a symmetry-related ina ligand [Sm—O = 2.389 (4)–2.791 (4) Å], giving a distorted monocapped square anti­prismatic geometry. The Ag^I^ ion is three-coordinated in a T-shaped geometry involving two ina N-donor atoms [Ag—N = 2.181 (6) and 2.185 (5) Å] and a bridging oxalate O-donor atom [Ag—O = 2.620 (4) Å]. The three-dimensional heterometallic Sm—Ag coordination polymer, having a unique (3,4,6)-connected five-nodal net topology, is constructed from two-dimensional samarium–oxalate layers and pillared Ag(ina)_2_ subunits. Inter­molecular water–carboxyl­ate O—H⋯O hydrogen-bonding inter­actions are also present.

## Related literature

For microporous metal-organic framework (MMOF) compounds, see: Sun *et al.* (2006 ▶); Wu & Lin (2005 ▶); Cho *et al.* (2006 ▶). For isonicotinic acid-heterometallic compounds, see: Cai *et al.* (2009 ▶); Gu & Xue (2006 ▶, 2007 ▶); Ma *et al.* (2009 ▶). For topological studies, see: Blatov *et al.* (2000 ▶); Blatov & Shevchenko (2006 ▶).
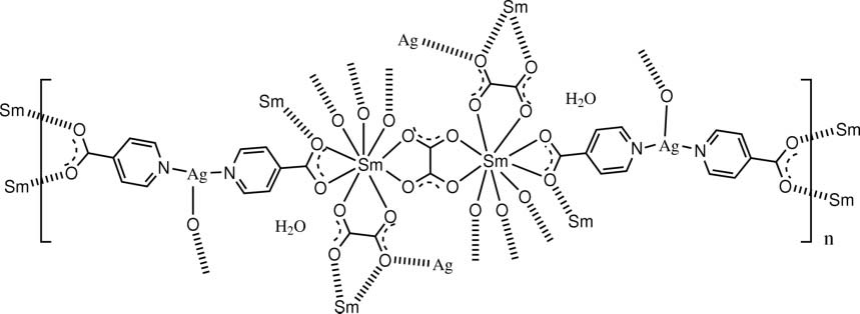


## Experimental

### 

#### Crystal data

[AgSm(C_6_H_4_NO_2_)_2_(C_2_O_4_)]·H_2_O
*M* *_r_* = 608.47
Monoclinic, 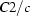

*a* = 22.0484 (18) Å
*b* = 9.2372 (8) Å
*c* = 17.1137 (14) Å
β = 108.123 (1)°
*V* = 3312.6 (5) Å^3^
*Z* = 8
Mo *K*α radiation
μ = 4.75 mm^−1^
*T* = 298 K
0.30 × 0.23 × 0.18 mm

#### Data collection

Bruker SMART APEX CCD-detector diffractometer
Absorption correction: multi-scan (*SADABS*; Sheldrick, 2004 ▶) *T*_min_ = 0.330, *T*_max_ = 0.482
8766 measured reflections
3240 independent reflections
2789 reflections with *I* > 2σ(*I*)
*R*_int_ = 0.057

#### Refinement

*R*[*F* ^2^ > 2σ(*F* ^2^)] = 0.037
*wR*(*F* ^2^) = 0.097
*S* = 1.08
3240 reflections
244 parameters
H-atom parameters constrained
Δρ_max_ = 1.43 e Å^−3^
Δρ_min_ = −1.50 e Å^−3^

### 

Data collection: *SMART* (Bruker, 2004 ▶); cell refinement: *SAINT* (Bruker, 2004 ▶); data reduction: *SAINT*; program(s) used to solve structure: *SHELXS97* (Sheldrick, 2008 ▶); program(s) used to refine structure: *SHELXL97* (Sheldrick, 2008 ▶); molecular graphics: *SHELXTL* (Sheldrick, 2008 ▶); software used to prepare material for publication: *SHELXTL*.

## Supplementary Material

Crystal structure: contains datablocks I, global. DOI: 10.1107/S1600536809054208/zs2014sup1.cif

Structure factors: contains datablocks I. DOI: 10.1107/S1600536809054208/zs2014Isup2.hkl

Additional supplementary materials:  crystallographic information; 3D view; checkCIF report

## Figures and Tables

**Table 1:** Hydrogen-bond geometry (Å, °)

*D*—H⋯*A*	*D*—H	H⋯*A*	*D*⋯*A*	*D*—H⋯*A*
O1*W*—H1*W*⋯O8^i^	0.86	2.16	2.960 (8)	156
O1*W*—H2*W*⋯O3	0.86	2.31	2.915 (7)	128

Symmetry code: (i) 
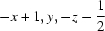
.

**Fig. 1.**
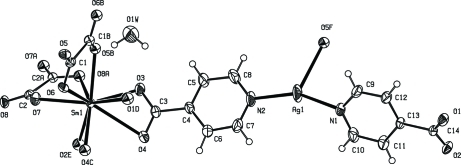
Displacement ellipsoid plot (40% probability level) of the title compound, with atom numbering scheme for non-H atoms. For symmetry-generated atoms, codes are: (A) -x + 1, y, -z - 1/2; (B) -x + 1, -y, -z; (C) -x + 1, -y + 1, -z; (D) -x + 1/2, y - 1/2,-z + 1/2; (E) x + 1/2,-y + 3/2, z - 1/2; (F) x - 1/2, y + 1/2, z].

**Fig. 2.**
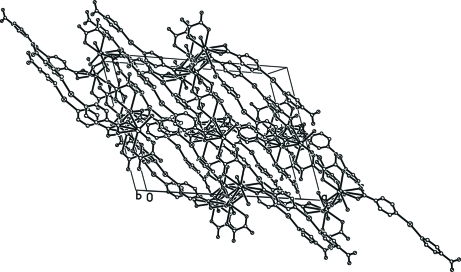
The three-dimensional pillar-layered structure in a packing diagram of the title compound, with H atoms omitted for clarity. Hydrogen bonds are shown as dashed lines.

## supplementary crystallographic information

### Comment

Microporous metal-organic frameworks (MMOFs) are of great current interest in view of their fascinating structural topologies and potential applications, e.g. in small molecule gas storage, separation and catalysis (Sun *et al.*, 2006; Wu *et al.*, 2005; Cho *et al.*, 2006). However, most of the works have so far focused on the assembly of 3d block metals with organic ligands as linkers and many 3d-4f heterometallic MOFs have also been reported. However, the 4d-4f heterometallic compounds based on the isonicotinic acid (ina) ligand have received less attention (Gu *et al.*, 2006; Gu *et al.*, 2007; Ma *et al.*, 2009; Cai *et al.*, 2009). The preparation of 4d-4f MMOFs has certain difficulties because of the high coordination number of 4f block metals, which frequently leads to interpenetration and consequently results in a decrease of the pore size or the MMOF may even become nonporous. Therefore the selection of the organic ligands becomes a key point in the preparation of 4d-4f heterometallic MMOFs. Herein, we report the structure of the title compound {[SmAg(C_6_H_4_NO_2_)_2_(C_2_O_4_)].H_2_O}_n_ (I) involving Sm^III^, Ag^I^ and the organic nicotinate and oxalate ligands, which has a microporous structure.

In the title compound (Fig. 1), the asymmetric unit contains one Sm^III^ ion, one Ag^I^ ion, two unique isonicotinate (ina) ligands, two half oxalate (ox) ligands [one on an inversion centre (associated with O5 and O6), the other on a two-fold axis (associated with O7 and O8)] and one uncoordinated water molecule of solvation (O1W). The central Sm^III^ ion is nine-coordinate with four O-donor atoms from separate bidentate bridging ox ligands and five O-donors from the two ina ligands (both bidentate) [Sm—O bond length range, 2.389 (4)–2.791 (4) Å], giving a distorted monocapped square antiprismatic stereochemistry. The three-coordinate Ag^I^ion is surrounded by two N-donor atoms from the two ina ligands [Ag–N, 2.181 (6), 2.185 (5) Å] and one O atom from a bridging oxalate ligand giving a T-shaped coordination geometry. The Ag—O bond [2.620 (4) Å] is long but this and the N—Ag—N angle [154.3 (2)°] are similar to those found in other Ag^I^ complexes having T-shaped configurations. The oxalate ligands bridge Sm centers to form a two-dimensional lanthanide-oxalate layered network. In the packing arrangement of the title compound, 'linear' N–Ag–N linkages play an important role in connecting the adjacent two-dimensional layers, forming a three-dimensional pillar-layered coordination polymer with microporous structures. Topological studies performed using the software package TOPOS 4.0 (Blatov & Shevchenko, 2006; Blatov *et al.*, 2000) reveal that this topology is a unique five-nodal (3,4,6)-connected net. The water molecule of solvation also gives O–H···O hydrogen-bonding interactions with oxalate and isonicotinate O acceptors (Table 1).

### Experimental

A mixture of isonicotinic acid (0.0615 g), Sm(NO_3_)_3_.6H_2_O (0.114 g), AgNO_3_ (0.051 g, 0.3mmol), oxalic acid dihydrate (0.037 g, 0.3mmol) and water (10 ml) was heated at 430 K for 72 h in a 23 ml Teflon-lined stainless-steel autoclave and then cooled to room temperature at a rate of 278° per hour. Colourless prismatic crystals were collected, washed with water three times and dried in air.

### Refinement

All H atoms were placed at calculated positions and were treated as riding on the parent C atoms with C—H = 0.93 |%A and O—H = 0.86 Å, and with *U*_iso_(H) = 1.2 or 1.5 *U*_eq_(C, O). The H atoms of the water molecule (O1W) were found from the difference Fourier maps and fixed using AFIX within SHELXL97 (Sheldrick, 2008).

### Crystal data

**Table:**

[AgSm(C_6_H_4_NO_2_)_2_(C_2_O_4_)]·H_2_O	*F*(000) = 2312
*M**_r_* = 608.47	*D*_x_ = 2.440 Mg m^−^^3^
Monoclinic, *C*2/*c*	Mo *K*α radiation, λ = 0.71073 Å
Hall symbol: -C 2yc	Cell parameters from 3815 reflections
*a* = 22.0484 (18) Å	θ = 2.4–27.8°
*b* = 9.2372 (8) Å	µ = 4.75 mm^−^^1^
*c* = 17.1137 (14) Å	*T* = 298 K
β = 108.123 (1)°	Prism, colorless
*V* = 3312.6 (5) Å^3^	0.30 × 0.23 × 0.18 mm
*Z* = 8	

### Data collection

**Table:**

Bruker SMART APEX CCD-detector diffractometer	3240 independent reflections
Radiation source: fine-focus sealed tube	2789 reflections with *I* > 2σ(*I*)
graphite	*R*_int_ = 0.057
ω scans	θ_max_ = 26.0°, θ_min_ = 1.9°
Absorption correction: multi-scan (*SADABS*; Sheldrick, 2004)	*h* = −27→27
*T*_min_ = 0.330, *T*_max_ = 0.482	*k* = −11→11
8766 measured reflections	*l* = −16→21

### Refinement

**Table:**

Refinement on *F*^2^	Primary atom site location: structure-invariant direct methods
Least-squares matrix: full	Secondary atom site location: difference Fourier map
*R*[*F*^2^ > 2σ(*F*^2^)] = 0.037	Hydrogen site location: inferred from neighbouring sites
*wR*(*F*^2^) = 0.097	H-atom parameters constrained
*S* = 1.08	*w* = 1/[σ^2^(*F*_o_^2^) + (0.0301*P*)^2^ + 2.6943*P*] where *P* = (*F*_o_^2^ + 2*F*_c_^2^)/3
3240 reflections	(Δ/σ)_max_ < 0.001
244 parameters	Δρ_max_ = 1.43 e Å^−^^3^
0 restraints	Δρ_min_ = −1.50 e Å^−^^3^

### Special details

**Table:**

Geometry. All esds (except the esd in the dihedral angle between two l.s. planes) are estimated using the full covariance matrix. The cell esds are taken into account individually in the estimation of esds in distances, angles and torsion angles; correlations between esds in cell parameters are only used when they are defined by crystal symmetry. An approximate (isotropic) treatment of cell esds is used for estimating esds involving l.s. planes.
Refinement. Refinement of F^2^ against ALL reflections. The weighted R-factor wR and goodness of fit S are based on F^2^, conventional R-factors R are based on F, with F set to zero for negative F^2^. The threshold expression of F^2^ > 2sigma(F^2^) is used only for calculating R-factors(gt) etc. and is not relevant to the choice of reflections for refinement. R-factors based on F^2^ are statistically about twice as large as those based on F, and R- factors based on ALL data will be even larger.

### Fractional atomic coordinates and isotropic or equivalent isotropic displacement parameters (Å^2^)

**Table:**

	*x*	*y*	*z*	*U*_iso_*/*U*_eq_	
Ag1	0.17077 (3)	0.56369 (8)	0.13601 (4)	0.0685 (2)	
C1	0.5352 (2)	0.0142 (5)	0.0244 (3)	0.0262 (11)	
C2	0.5364 (2)	0.2525 (5)	−0.2303 (3)	0.0240 (11)	
C3	0.3774 (2)	0.4799 (6)	−0.0550 (3)	0.0278 (11)	
C4	0.3236 (3)	0.5115 (6)	−0.0213 (3)	0.0317 (12)	
C5	0.2693 (4)	0.4269 (10)	−0.0417 (6)	0.082 (3)	
H5	0.2627	0.3567	−0.0825	0.098*	
C6	0.3283 (3)	0.6170 (8)	0.0352 (5)	0.0520 (18)	
H6	0.3623	0.6817	0.0473	0.062*	
C7	0.2830 (3)	0.6284 (8)	0.0745 (4)	0.0527 (19)	
H7	0.2884	0.6992	0.1148	0.063*	
C8	0.2246 (4)	0.4474 (10)	−0.0008 (6)	0.086 (3)	
H8	0.1878	0.3910	−0.0155	0.103*	
C9	0.1123 (4)	0.5823 (7)	0.2779 (5)	0.064 (2)	
H9	0.1160	0.4822	0.2760	0.077*	
C10	0.1345 (3)	0.8035 (7)	0.2388 (4)	0.0437 (16)	
H10	0.1547	0.8615	0.2101	0.052*	
C11	0.1016 (3)	0.8711 (7)	0.2855 (4)	0.0408 (15)	
H11	0.0985	0.9714	0.2866	0.049*	
C12	0.0803 (3)	0.6383 (7)	0.3267 (4)	0.0490 (18)	
H12	0.0630	0.5773	0.3574	0.059*	
C13	0.0734 (3)	0.7849 (6)	0.3305 (3)	0.0255 (11)	
C14	0.0374 (2)	0.8525 (6)	0.3833 (3)	0.0262 (11)	
N1	0.1388 (2)	0.6622 (6)	0.2325 (3)	0.0420 (13)	
N2	0.2324 (3)	0.5447 (6)	0.0585 (3)	0.0492 (14)	
O1	0.0347 (2)	0.7806 (4)	0.4442 (2)	0.0353 (9)	
O2	0.01455 (19)	0.9765 (4)	0.3628 (2)	0.0337 (9)	
O3	0.37193 (18)	0.3777 (5)	−0.1045 (2)	0.0388 (10)	
O4	0.42816 (17)	0.5524 (4)	−0.0283 (2)	0.0316 (8)	
O5	0.56218 (17)	−0.0747 (4)	0.0790 (2)	0.0286 (8)	
O6	0.55987 (17)	0.1263 (4)	0.0070 (2)	0.0307 (8)	
O7	0.55695 (18)	0.2331 (4)	−0.1546 (2)	0.0332 (9)	
O8	0.56874 (17)	0.2685 (4)	−0.2785 (2)	0.0309 (8)	
O1W	0.2965 (3)	0.1982 (6)	−0.2401 (4)	0.0729 (17)	
H1W	0.3311	0.2196	−0.2506	0.109*	
H2W	0.2925	0.2570	−0.2031	0.109*	
Sm1	0.487549 (12)	0.31384 (3)	−0.073228 (15)	0.02264 (12)	

### Atomic displacement parameters (Å^2^)

**Table:**

	*U*^11^	*U*^22^	*U*^33^	*U*^12^	*U*^13^	*U*^23^
Ag1	0.0488 (3)	0.1075 (6)	0.0651 (4)	−0.0103 (3)	0.0408 (3)	−0.0402 (4)
C1	0.037 (3)	0.022 (3)	0.025 (3)	−0.004 (2)	0.017 (2)	−0.005 (2)
C2	0.036 (3)	0.016 (3)	0.027 (3)	0.000 (2)	0.020 (2)	0.000 (2)
C3	0.027 (3)	0.035 (3)	0.022 (3)	0.002 (2)	0.010 (2)	0.003 (2)
C4	0.029 (3)	0.041 (3)	0.031 (3)	−0.004 (2)	0.017 (2)	−0.003 (2)
C5	0.063 (5)	0.109 (7)	0.099 (7)	−0.046 (5)	0.061 (5)	−0.083 (6)
C6	0.036 (3)	0.056 (4)	0.071 (5)	−0.012 (3)	0.027 (3)	−0.032 (4)
C7	0.037 (3)	0.067 (5)	0.057 (4)	−0.006 (3)	0.020 (3)	−0.037 (4)
C8	0.068 (5)	0.110 (8)	0.109 (7)	−0.049 (5)	0.070 (5)	−0.069 (6)
C9	0.090 (6)	0.031 (4)	0.104 (6)	−0.011 (3)	0.080 (5)	−0.017 (4)
C10	0.048 (4)	0.054 (4)	0.042 (4)	0.001 (3)	0.032 (3)	0.006 (3)
C11	0.056 (4)	0.033 (3)	0.047 (4)	0.011 (3)	0.035 (3)	0.016 (3)
C12	0.072 (5)	0.028 (3)	0.072 (5)	−0.007 (3)	0.059 (4)	−0.005 (3)
C13	0.031 (3)	0.030 (3)	0.018 (2)	0.003 (2)	0.013 (2)	0.001 (2)
C14	0.030 (3)	0.027 (3)	0.028 (3)	−0.001 (2)	0.017 (2)	−0.005 (2)
N1	0.045 (3)	0.049 (3)	0.044 (3)	−0.006 (2)	0.031 (3)	−0.018 (2)
N2	0.045 (3)	0.066 (4)	0.051 (3)	−0.001 (3)	0.034 (3)	−0.016 (3)
O1	0.052 (3)	0.033 (2)	0.030 (2)	0.0029 (18)	0.0265 (19)	0.0004 (17)
O2	0.050 (2)	0.028 (2)	0.030 (2)	0.0086 (17)	0.0228 (18)	−0.0002 (16)
O3	0.035 (2)	0.050 (3)	0.037 (2)	−0.0027 (18)	0.0180 (18)	−0.015 (2)
O4	0.0268 (19)	0.036 (2)	0.035 (2)	−0.0004 (17)	0.0138 (16)	0.0022 (17)
O5	0.035 (2)	0.0238 (19)	0.028 (2)	−0.0042 (15)	0.0108 (16)	0.0055 (15)
O6	0.030 (2)	0.026 (2)	0.040 (2)	−0.0057 (16)	0.0180 (17)	0.0028 (17)
O7	0.039 (2)	0.041 (2)	0.025 (2)	0.0037 (18)	0.0167 (17)	0.0006 (17)
O8	0.033 (2)	0.035 (2)	0.033 (2)	0.0030 (16)	0.0217 (17)	0.0026 (17)
O1W	0.058 (3)	0.081 (4)	0.080 (4)	−0.006 (3)	0.022 (3)	−0.011 (3)
Sm1	0.02976 (18)	0.02037 (18)	0.02401 (18)	−0.00243 (10)	0.01742 (13)	−0.00082 (9)

### Geometric parameters (Å, °)

**Table:**

Sm1—O3	2.507 (4)	N1—C9	1.331 (10)
Sm1—O4	2.791 (4)	N2—C8	1.326 (11)
Sm1—O6	2.463 (4)	N2—C7	1.314 (10)
Sm1—O7	2.483 (4)	C1—C1^ii^	1.539 (7)
Sm1—O8^i^	2.489 (3)	C2—C2^i^	1.536 (7)
Sm1—O5^ii^	2.454 (4)	C3—C4	1.500 (8)
Sm1—O4^iii^	2.448 (4)	C4—C6	1.354 (9)
Sm1—O1^iv^	2.426 (4)	C4—C5	1.381 (11)
Sm1—O2^v^	2.389 (4)	C5—C8	1.388 (13)
Ag1—N1	2.185 (5)	C6—C7	1.371 (10)
Ag1—N2	2.181 (6)	C9—C12	1.353 (11)
Ag1—O5^vi^	2.620 (4)	C10—C11	1.384 (10)
O1—C14	1.253 (6)	C11—C13	1.383 (9)
O2—C14	1.257 (7)	C12—C13	1.367 (9)
O3—C3	1.249 (7)	C13—C14	1.511 (8)
O4—C3	1.262 (6)	C5—H5	0.9300
O5—C1	1.247 (6)	C6—H6	0.9300
O6—C1	1.248 (6)	C7—H7	0.9300
O7—C2	1.245 (6)	C8—H8	0.9300
O8—C2	1.256 (6)	C9—H9	0.9300
O1W—H2W	0.8600	C10—H10	0.9300
O1W—H1W	0.8600	C11—H11	0.9300
N1—C10	1.316 (9)	C12—H12	0.9300
			
O3—Sm1—O4	48.69 (12)	Sm1^i^—O8—C2	119.0 (3)
O3—Sm1—O6	136.35 (13)	H1W—O1W—H2W	108.00
O3—Sm1—O7	135.58 (11)	Ag1—N1—C9	120.9 (5)
O3—Sm1—O8^i^	70.76 (12)	Ag1—N1—C10	121.6 (4)
O3—Sm1—O5^ii^	77.95 (14)	C9—N1—C10	116.5 (6)
O3—Sm1—O4^iii^	122.00 (13)	Ag1—N2—C8	124.3 (6)
O1^iv^—Sm1—O3	75.17 (13)	C7—N2—C8	117.2 (7)
O2^v^—Sm1—O3	95.35 (14)	Ag1—N2—C7	118.4 (4)
O4—Sm1—O6	132.82 (10)	O5—C1—O6	125.7 (5)
O4—Sm1—O7	144.66 (11)	O6—C1—C1^ii^	116.9 (4)
O4—Sm1—O8^i^	106.61 (11)	O5—C1—C1^ii^	117.4 (4)
O4—Sm1—O5^ii^	118.67 (12)	O7—C2—O8	127.1 (5)
O4—Sm1—O4^iii^	73.95 (12)	O8—C2—C2^i^	116.2 (4)
O1^iv^—Sm1—O4	66.68 (11)	O7—C2—C2^i^	116.7 (4)
O2^v^—Sm1—O4	72.02 (12)	O3—C3—O4	122.3 (5)
O6—Sm1—O7	72.32 (12)	O3—C3—C4	119.1 (5)
O6—Sm1—O8^i^	118.75 (12)	O4—C3—C4	118.4 (5)
O5^ii^—Sm1—O6	65.99 (12)	C3—C4—C5	121.3 (6)
O4^iii^—Sm1—O6	75.07 (12)	C3—C4—C6	121.4 (6)
O1^iv^—Sm1—O6	71.50 (13)	C5—C4—C6	117.1 (7)
O2^v^—Sm1—O6	127.96 (13)	C4—C5—C8	119.4 (8)
O7—Sm1—O8^i^	64.94 (12)	C4—C6—C7	120.0 (7)
O5^ii^—Sm1—O7	93.02 (12)	N2—C7—C6	123.6 (7)
O4^iii^—Sm1—O7	94.90 (12)	N2—C8—C5	122.3 (8)
O1^iv^—Sm1—O7	143.81 (13)	N1—C9—C12	123.8 (6)
O2^v^—Sm1—O7	72.64 (13)	N1—C10—C11	124.0 (6)
O5^ii^—Sm1—O8^i^	74.50 (11)	C10—C11—C13	118.0 (6)
O4^iii^—Sm1—O8^i^	146.76 (12)	C9—C12—C13	119.7 (6)
O1^iv^—Sm1—O8^i^	136.79 (13)	C11—C13—C14	120.4 (5)
O2^v^—Sm1—O8^i^	77.62 (12)	C12—C13—C14	121.7 (5)
O4^iii^—Sm1—O5^ii^	135.56 (11)	C11—C13—C12	117.9 (6)
O1^iv^—Sm1—O5^ii^	73.05 (12)	O1—C14—O2	126.6 (5)
O2^v^—Sm1—O5^ii^	151.99 (11)	O2—C14—C13	116.7 (4)
O1^iv^—Sm1—O4^iii^	75.03 (13)	O1—C14—C13	116.6 (5)
O2^v^—Sm1—O4^iii^	70.96 (12)	C4—C5—H5	120.00
O1^iv^—Sm1—O2^v^	132.02 (12)	C8—C5—H5	120.00
N1—Ag1—N2	154.3 (2)	C7—C6—H6	120.00
O5^vi^—Ag1—N1	90.71 (15)	C4—C6—H6	120.00
O5^vi^—Ag1—N2	113.85 (17)	N2—C7—H7	118.00
Sm1^vii^—O1—C14	140.0 (3)	C6—C7—H7	118.00
Sm1^viii^—O2—C14	138.3 (3)	C5—C8—H8	119.00
Sm1—O3—C3	99.0 (3)	N2—C8—H8	119.00
Sm1—O4—C3	85.4 (3)	N1—C9—H9	118.00
Sm1—O4—Sm1^iii^	106.05 (13)	C12—C9—H9	118.00
Sm1^iii^—O4—C3	156.5 (3)	C11—C10—H10	118.00
Sm1^ii^—O5—C1	117.4 (3)	N1—C10—H10	118.00
Ag1^ix^—O5—C1	97.2 (3)	C10—C11—H11	121.00
Sm1^ii^—O5—Ag1^ix^	143.05 (16)	C13—C11—H11	121.00
Sm1—O6—C1	117.5 (3)	C9—C12—H12	120.00
Sm1—O7—C2	116.9 (3)	C13—C12—H12	120.00
			
O8^i^—Sm1—O6—C1	−72.4 (4)	O3^i^—Sm1^i^—O8—C2	164.2 (4)
O5^ii^—Sm1—O6—C1	−17.8 (3)	O4^i^—Sm1^i^—O8—C2	130.7 (3)
O4^iii^—Sm1—O6—C1	140.3 (4)	O5^vi^—Ag1—N1—C9	47.9 (5)
O1^iv^—Sm1—O6—C1	61.4 (3)	N1—Ag1—O5^vi^—C1^vi^	95.8 (3)
O2^v^—Sm1—O6—C1	−169.2 (3)	N2—Ag1—O5^vi^—C1^vi^	−76.5 (3)
O3—Sm1—O7—C2	17.4 (4)	O5^vi^—Ag1—N1—C10	−120.1 (5)
O4—Sm1—O7—C2	−61.6 (4)	N1—Ag1—N2—C7	7.2 (8)
O6—Sm1—O7—C2	156.9 (4)	N1—Ag1—N2—C8	−177.3 (6)
O8^i^—Sm1—O7—C2	21.9 (3)	O5^vi^—Ag1—N2—C7	169.2 (5)
O5^ii^—Sm1—O7—C2	93.3 (3)	O5^vi^—Ag1—N2—C8	−15.3 (7)
O3^vii^—Sm1^vii^—O1—C14	−84.5 (6)	N2—Ag1—N1—C9	−148.6 (6)
O4^vii^—Sm1^vii^—O1—C14	−33.5 (5)	N2—Ag1—N1—C10	43.5 (7)
O6^vii^—Sm1^vii^—O1—C14	124.1 (6)	Sm1^vii^—O1—C14—C13	161.8 (4)
O7^vii^—Sm1^vii^—O1—C14	122.7 (5)	Sm1^vii^—O1—C14—O2	−16.4 (9)
O4^viii^—Sm1^vii^—O1—C14	45.2 (5)	Sm1^viii^—O2—C14—O1	26.0 (9)
O4^vii^—Sm1^viii^—O2—C14	26.1 (5)	Sm1^viii^—O2—C14—C13	−152.2 (4)
O3^viii^—Sm1^viii^—O2—C14	−95.9 (5)	Sm1—O3—C3—C4	−151.9 (4)
O4^viii^—Sm1^viii^—O2—C14	−52.6 (5)	Sm1—O3—C3—O4	23.7 (5)
O6^viii^—Sm1^viii^—O2—C14	78.2 (5)	Sm1—O4—C3—O3	−21.0 (5)
O7^viii^—Sm1^viii^—O2—C14	127.8 (5)	Sm1^iii^—O4—C3—C4	33.8 (11)
O4—Sm1—O3—C3	−11.8 (3)	Sm1^iii^—O4—C3—O3	−141.8 (7)
O6—Sm1—O3—C3	101.1 (4)	Sm1—O4—C3—C4	154.7 (4)
O7—Sm1—O3—C3	−142.7 (3)	Sm1^ii^—O5—C1—O6	−163.5 (4)
O8^i^—Sm1—O3—C3	−147.0 (3)	Ag1^ix^—O5—C1—C1^ii^	−175.3 (3)
O5^ii^—Sm1—O3—C3	135.4 (3)	Ag1^ix^—O5—C1—O6	3.0 (5)
O4^iii^—Sm1—O3—C3	−1.3 (4)	Sm1^ii^—O5—C1—C1^ii^	18.3 (5)
O1^iv^—Sm1—O3—C3	60.0 (3)	Sm1—O6—C1—O5	−162.4 (4)
O2^v^—Sm1—O3—C3	−72.1 (3)	Sm1—O6—C1—C1^ii^	15.9 (5)
O3—Sm1—O4—C3	11.6 (3)	Sm1—O7—C2—O8	153.3 (4)
O6—Sm1—O4—C3	−108.4 (3)	Sm1—O7—C2—C2^i^	−28.7 (5)
O7—Sm1—O4—C3	125.4 (3)	Sm1^i^—O8—C2—C2^i^	4.0 (5)
O8^i^—Sm1—O4—C3	55.5 (3)	Sm1^i^—O8—C2—O7	−177.9 (4)
O5^ii^—Sm1—O4—C3	−25.6 (3)	Ag1—N1—C9—C12	−166.4 (6)
O4^iii^—Sm1—O4—C3	−159.2 (3)	C10—N1—C9—C12	2.2 (11)
O1^iv^—Sm1—O4—C3	−78.9 (3)	Ag1—N1—C10—C11	164.8 (5)
O2^v^—Sm1—O4—C3	126.1 (3)	C9—N1—C10—C11	−3.7 (10)
O3—Sm1—O4—Sm1^iii^	170.7 (2)	Ag1—N2—C8—C5	−171.7 (7)
O6—Sm1—O4—Sm1^iii^	50.8 (2)	Ag1—N2—C7—C6	173.7 (6)
O7—Sm1—O4—Sm1^iii^	−75.4 (2)	C7—N2—C8—C5	3.8 (12)
O8^i^—Sm1—O4—Sm1^iii^	−145.32 (13)	C8—N2—C7—C6	−2.1 (11)
O5^ii^—Sm1—O4—Sm1^iii^	133.58 (13)	O6—C1—C1^ii^—O6^ii^	180.0 (4)
O4^iii^—Sm1—O4—Sm1^iii^	0.02 (14)	O5—C1—C1^ii^—O6^ii^	−1.6 (7)
O1^iv^—Sm1—O4—Sm1^iii^	80.28 (15)	O6—C1—C1^ii^—O5^ii^	1.6 (7)
O2^v^—Sm1—O4—Sm1^iii^	−74.72 (14)	O5—C1—C1^ii^—O5^ii^	180.0 (4)
O3^iii^—Sm1^iii^—O4—Sm1	8.21 (18)	O7—C2—C2^i^—O8^i^	16.8 (6)
O4^iii^—Sm1^iii^—O4—Sm1	0.00 (11)	O8—C2—C2^i^—O7^i^	16.8 (6)
O6^iii^—Sm1^iii^—O4—Sm1	143.96 (15)	O7—C2—C2^i^—O7^i^	−161.5 (4)
O7^iii^—Sm1^iii^—O4—Sm1	−145.82 (12)	O8—C2—C2^i^—O8^i^	−164.9 (4)
O2^iv^—Sm1^iii^—O4—Sm1	−76.09 (14)	O3—C3—C4—C6	177.1 (6)
O1^v^—Sm1^iii^—O4—Sm1	69.54 (13)	O3—C3—C4—C5	2.3 (9)
O3^iii^—Sm1^iii^—O4—C3	125.3 (9)	O4—C3—C4—C6	1.3 (8)
O4^iii^—Sm1^iii^—O4—C3	117.0 (9)	O4—C3—C4—C5	−173.5 (6)
O6^iii^—Sm1^iii^—O4—C3	−99.0 (9)	C6—C4—C5—C8	−3.7 (12)
O7^iii^—Sm1^iii^—O4—C3	−28.8 (9)	C3—C4—C6—C7	−169.6 (6)
O2^iv^—Sm1^iii^—O4—C3	41.0 (9)	C3—C4—C5—C8	171.3 (7)
O1^v^—Sm1^iii^—O4—C3	−173.4 (9)	C5—C4—C6—C7	5.5 (10)
O3^ii^—Sm1^ii^—O5—C1	136.2 (4)	C4—C5—C8—N2	−0.9 (14)
O4^ii^—Sm1^ii^—O5—C1	108.6 (3)	C4—C6—C7—N2	−2.7 (11)
O6^ii^—Sm1^ii^—O5—C1	−18.6 (3)	N1—C9—C12—C13	0.6 (12)
O7^ii^—Sm1^ii^—O5—C1	−87.7 (3)	N1—C10—C11—C13	2.3 (10)
O3—Sm1—O6—C1	19.3 (4)	C10—C11—C13—C12	0.6 (9)
O4—Sm1—O6—C1	89.9 (4)	C10—C11—C13—C14	179.4 (5)
O7—Sm1—O6—C1	−119.4 (4)	C9—C12—C13—C11	−2.0 (10)
O6^i^—Sm1^i^—O8—C2	−62.8 (4)	C9—C12—C13—C14	179.3 (6)
O7^i^—Sm1^i^—O8—C2	−12.5 (3)	C11—C13—C14—O2	25.2 (8)
O2^v^—Sm1—O7—C2	−62.2 (3)	C12—C13—C14—O1	25.6 (8)
O4^iii^—Sm1—O7—C2	−130.5 (3)	C12—C13—C14—O2	−156.1 (6)
O1^iv^—Sm1—O7—C2	158.3 (3)	C11—C13—C14—O1	−153.1 (6)

Symmetry codes: (i) −*x*+1, *y*, −*z*−1/2; (ii) −*x*+1, −*y*, −*z*; (iii) −*x*+1, −*y*+1, −*z*; (iv) −*x*+1/2, *y*−1/2, −*z*+1/2; (v) *x*+1/2, −*y*+3/2, *z*−1/2; (vi) *x*−1/2, *y*+1/2, *z*; (vii) −*x*+1/2, *y*+1/2, −*z*+1/2; (viii) *x*−1/2, −*y*+3/2, *z*+1/2; (ix) *x*+1/2, *y*−1/2, *z*.

### Hydrogen-bond geometry (Å, °)

**Table:**

*D*—H···*A*	*D*—H	H···*A*	*D*···*A*	*D*—H···*A*
O1W—H1W···O8^i^	0.86	2.16	2.960 (8)	156
O1W—H2W···O3	0.86	2.31	2.915 (7)	128

Symmetry codes: (i) −*x*+1, *y*, −*z*−1/2.
